# A Two-Sided Lockdown? Social Class Variations in the Implementation of Homeschooling During the COVID-19 Lockdown

**DOI:** 10.3389/fpsyg.2021.670722

**Published:** 2021-10-27

**Authors:** Camille Sanrey, Sébastien Goudeau, Arnaud Stanczak, Céline Darnon

**Affiliations:** ^1^CeRCA – CNRS UMR 7295, Université de Poitiers, Poitiers, France; ^2^LPC, Université de Strasbourg, Strasbourg, France; ^3^LAPSCO – CNRS UMR 6024, Université Clermont Auvergne, Clermont-Ferrand, France

**Keywords:** COVID-19, lockdown, homeschool, parental practices, social inequalities in education

## Abstract

The COVID-19 pandemic deeply affected how schools and families functioned through most of 2020. In particular, school closures meant parents took on a more central role in their children’s learning. This study analyzed social class variations in the quantity and quality of homeschooling during the lockdown. Through an online questionnaire, 360 parents reported (1) their digital equipment and use, (2) the perceptions of their ability to homeschool their children, (3) how they handled homeschooling and (4) the extent to which they supported other activities considered more or less “profitable” from an educational point of view (e.g., reading, watching television). A social position index was used as a proxy of social class. The results indicated that all parents were highly involved in setting up homeschooling and that the lower the parents’ social position, the more they spent time homeschooling their children. However, in line with the digital divide literature, the lower the parents’ social position, the lower the digital equipment and the less the parents felt capable of homeschooling. Finally, the higher the social position of the families, the more children spent time doing activities considered to be “educationally profitable,” and the less they spent time doing “unprofitable activities.” Thus, even if all parents were highly involved in homeschooling, higher social position parents were better equipped both materially and psychologically to face the challenge of homeschooling. The long-term impact of these processes on the perpetuation of social class inequalities are discussed.

## A Two-Sided Lockdown? Social Class Variations in the Implementation of Homeschooling During the COVID-19 Lockdown

In the beginning of 2020, the COVID-19 pandemic deeply affected the functioning of societies around the world. In particular, the first wave of the pandemic led many countries to close schools, impacting hundreds of millions of learners (UNESCO, 2020). More recently, highly contagious variants of the virus have emerged, forcing a new period of school closures in many countries worldwide. This situation places a heavy responsibility on parents (Lee et al., 2021) and recent studies have documented that the lockdown has increased the risk of parental stress (Griffith, 2020; Spinelli et al., 2020) and parenting-related exhaustion (Marchetti et al., 2020). In addition, higher levels of depression and anxiety have been observed among parents and children than in normal times (Zhao et al., 2020). All these difficulties, due in large part to the fact parents had to homeschool their children (Thorell et al., 2021), are particularly true for working-class parents (Goudeau et al., 2021; Parolin and Lee, 2021). The goal of the present paper is to document social class variations in the implementation of homeschooling in France during spring 2020.

Social class is a powerful context of life and socialization associated with diverse material, cultural, and psychological resources that constitute (dis)advantages for many aspects of schooling (Stephens et al., 2012; Goudeau et al., 2017). More precisely, research has highlighted the existence of “divides,” which can be particularly problematic when schools are closed. These divides concern both digital equipment and use (i.e., “digital divide,” Zhang, 2015; Harris et al., 2017), and cultural practices that appear to be more or less “profitable” in terms of educational outcomes (e.g., Bourdieu and Passeron, 1990; Lareau, 2003; Gaddis, 2013), as well as parental perceptions of their ability to homeschool their children (Tazouti and Jarlégan, 2016).

The digital divide refers to the fact that social class is a strong and recurrent predictor of digital access, skills, and use of digital tools (e.g., Harris et al., 2017; Anderson and Kumar, 2019). Indeed, upper-middle-class families^1^ not only live in larger houses and have more available space in which to study, they also have better digital equipment. Although the digital divide in access to digital tools has decreased over time, working-class families are still less equipped than upper-middle-class families and, thus, are more likely to be partially or totally excluded from the digital world (e.g., Cruz-Jesus et al., 2016). For example, in the United States in 2019, 41% of working-class families did not own a computer, compared to 8% of upper-middle-class families (Vogels, 2021). In addition to access to digital equipment, disparities in digital use also exist (Yates et al., 2015: Harris et al., 2017). For example, working-class families are more likely to use digital tools for entertainment than upper-middle-class families (e.g., video games; Bonfadelli, 2002; Harris et al., 2017), who are more likely to use digital tools for work or educational purposes (Robinson and Schulz, 2013; Harris et al., 2017).

In addition, as mentioned above, the school system plays an important role in reproducing social inequalities (Bourdieu and Passeron, 1990), notably by promoting practices, languages and way of being that are more in line with those developed in upper-class families than in working-class families. Thus, beyond the digital divide, working-class families usually have less familiarity with the academic knowledge and skills expected and valued in school compared to upper-middle-class families (Lamont and Lareau, 1988; Goudeau and Croizet, 2017). Consequently, working-class families are less likely to engage in cultural practices that match school curriculum (e.g., reading stories to children, visiting museums, Bernstein, 1974; Lareau, 2003; Gaddis, 2013). This lower familiarity toward academic knowledge and skills constitutes a disadvantage for working-class parents, who are likely to feel particularly challenged when they have to homeschool their children. Combined with other factors, such as negative stereotypes regarding one’s intelligence (e.g., Jury et al., 2017; Grigoryan et al., 2019), this lower familiarity with (academic) cultural capital may lead working-class parents into developing a poor sense of academic self-efficacy (Wiederkehr et al., 2015; Tazouti and Jarlégan, 2016). This poor self-efficacy may also be associated with a greater fear of academic failure for their children (see Wagner and Brahm, 2017).

Furthermore, because of these differences in cultural capital, upper-middle-class and working-class families may also differ in the nature of activities they supported among their children during the lockdown. Indeed, some activities (e.g., reading stories to their children) have the potential to increase students’ cultural capital (Lareau, 2003; Gaddis, 2013; Lahire, 2019). In contrast, other activities (e.g., watching television) are less “profitable” because beyond being less aligned with schools’ expectations and less valued by teachers, they are less likely to develop academic skills. Supporting this idea, working-class families have been shown to regulate their children’s television use to a lesser degree (Mentec and Plantard, 2014; Nikken and Jansz, 2014) and tend to watch less educative TV programs than upper-middle-class ones, further affecting their achievement (Sullivan, 2001). By supporting these activities, upper-middle-class parents provide their children with a cultural advantage that appears to be profitable for future academic success. Indeed, reading, practicing creative activities, and exercising are all activities that have been shown to be linked to cognitive development (e.g., Alvarez-Bueno et al., 2017) or academic achievement (e.g., Bus et al., 1995; Swanson et al., 2011; Gajda et al., 2017), whereas watching TV is negatively associated with achievement (e.g., Williams et al., 1982; Razel, 2001). Interestingly, the differential implementation of profitable and unprofitable activities at home is one of the reasons underlying the summer learning loss (i.e., the increase in the social class achievement gap during school breaks; Cooper et al., 1996; Alexander et al., 2007; Stewart et al., 2018).

## Overview and Hypotheses

In this paper, we seek to analyze how social class predicts the quantity and quality of homeschooling during the 2020 lockdown. More precisely, we examined social class variations on four main categories of outcomes: (1) digital equipment, (2) parents’ perceptions of their ability to homeschool their children and fear of their children’s academic failure, (3) implementation of homeschooling during school closure (e.g., duration of homeschooling, completion of exercises sent by the teachers), and (4) engagement in other profitable and unprofitable activities during school closure.

First, we hypothesize that the lower the parents’ social position, the lower their access to digital tools and the more they should use these tools for entertainment rather than for education. Second, although all families are expected to support homeschooling (e.g., do the exercises sent by the teachers), lower social position should be associated to lower levels of self-efficacy for homeschooling as well as a greater fear of academic failure. Finally, the likelihood to support profitable (vs. unprofitable) activities should increase (vs. decrease) with the parents’ social position.

## Method

### Participants

French parents of preschool- to elementary-school-aged children were invited to respond to an online questionnaire shared through personal, professional, and social networks in April 2020. The questionnaire was fully answered by a total of 360 parents (290 women, 68 men and 2 non-binary people; *M*_age_ = 37.70, SD = 5.10, min = 19, max = 53). Parents in this sample had on average two children (*M* = 2.07, SD = 0.84, min = 1, max = 6). Children were 164 girls (45.56%) and 196 boys, *M*_age_ = 6.32 years (SD = 2.13, *min* = 3, *max* = 11); 162 were enrolled in preschool (45.25%) and 196 were enrolled in elementary school (54.75%). Responses to all the questions were mandatory (except for the socio-demographic questions concerning the partner). Thus, there was no missing data. We used the *Social Position Index* (SPI) as a proxy of social class. This indicator is a standardized continuous variable, with mean = 100 and standard deviation = 30. It has been developed on large French databases in order to capture multiple dimensions linked to social class (e.g., educational attainment, parental education, material conditions, cultural capital, Rocher (2016), for a description of each possible social position values, see Supplementary Table 2). We assigned a social position index value to the respondent, as well as their partner, and kept the highest as a proxy of social class (Rocher, 2016).

### Measures

All measures and associated modalities are reported in Supplementary Table 1 in Supplementary Material.

#### Digital Equipment and Uses

Participants were asked to report whether they had (1) Internet access, (2) high-speed Internet access, (3) at least one computer at home, and (4) a printer (0 = *no*, 1 = *yes*). Participants who owned at least one computer were asked to indicate the number of owned computers and the number of users. Digital uses were assessed by asking participants about their frequency (from 1 = *never* to 4 = *several times a day*) and duration (average number of hours per week) of computer use for (1) leisure activities (e.g., watching movies), (2) work (e.g., professional email), and (3) schoolwork (e.g., information search). For each type of use, they were asked to answer for (a) normal times (i.e., before the lockdown) and (b) during the lockdown.

#### Perception of Homeschooling Ability

Perceived self-efficacy was measured using three items (α = 0.72) inspired from Tazouti and Jarlégan (2016) and adapted to the homeschooling context (e.g., “I am able to replace my child’s teacher during the lockdown” on scales ranging from 1 (*totally disagree*) to 7 (*totally agree*). Furthermore, fear of academic failure was measured using three items (α = 0.58) created to assess parental fear of failure concerning their child(ren)’s schooling (e.g., “I feel that my child will fall behind academically”; see SM for the entire scale), ranging from 1 (*totally disagree*) to 7 (*totally agree*).

#### Homeschooling Activities^2^

Participants were asked to indicate if they provided homework help in normal times (0 = *no*, 1 = *yes*) and, if so, its frequency per week (from 1 day to 7 days per week) as well as the average duration per day (from 1 = *less than 30 minutes a day* to 5 = *more than 3 hours a day*). They were then asked to indicate if they participated in homeschooling during the lockdown (0 = *no*, 1 = *yes*) and, if so, the frequency (i.e., number of days per week) and the average time per day (the same scale as for homework help, from 1 to 5). They then reported whether they received resources from their child’s teacher (0 = *no*; 1 = *yes*) and if they made their child do every exercise received (from 1 = *none* to 3 = *every one*). Participants were then asked whether they had made their child(ren) work on new concepts on their own initiative at least once (0 = *no*, 1 = *yes*) and if they knew of any additional resources (e.g., educational websites, educative television programs; 0 = *no*; 1 = *yes*). If so, they reported the frequency at which they used them (from 1 = *every day* to 5 = *never*).

#### Other (Profitable and Unprofitable) Activities During Lockdown

Participants indicated whether, during the lockdown, they had their child(ren) do some (1) creative activities (e.g., painting) and (2) sport activities (e.g., stretching; 0 = *no*, 1 = *yes*) and, if so, the frequency of these activities (from 1 = *every day* to 4 = *less than once a week*). Participants were also asked to specify the approximate time per week they^3^ spent reading books to their children and, if their children could already read, how long they spend reading by themselves (1) in normal times and (2) during the lockdown (from 1 = *less than 30 minutes a day* to 5 = *more than 3 hours a day*). Educationally unprofitable activities were assessed by asking participants to indicate the duration of television watching time (1) in normal times and (2) during the lockdown (from 1 = *less than 30 minutes a day* to 5 = *more than 3 hours a day*).

## Results

### Data Analyses

We analyzed the extent to which the social position index predicted the four categories of outcomes. Due to some normality and heteroscedasticity issues, we used robust regressions on continuous variables and logistical robust regressions on categorical variables (using the “robustbase” R package). It is worth noting that in further analyses, we computed covariates analyses. These covariates analyses are reported in Supplementary Tables 3, 4 (see Supplementary Material).

### Digital Equipment and Uses

The higher the social position, the higher the probability to own a computer, *Z* = 3.14, *p* = 0.002, OR = 1.03, 97.5% CI = [1.01; 1.05], the higher the probability to own a printer (marginal), *Z* = 1.70, *p* = 0.089, OR = 1.01, 97.5% CI = [0.99; 1.02], the higher the probability to have access to high-speed Internet, *Z* = 2.25, *p* = 0.025, OR = 1.01, 97.5% CI = [1.00; 1.02] and the higher the number of owned computers, *t*(358) = 5.30, *p* < 0.001, IRR = 1.01, 97.5% CI = [1.01; 1.02].

In normal times, the higher the social position, the higher the frequency of leisure use (marginal), *t*(341) = 1.85, *p* = 0.066, IRR = 1.01, 97.5% CI = [0.99; 1.02], and the higher the duration of leisure use, *t*(336) = 2.21, *p* = 0.028, IRR = 1.02, 97.5% CI = [1.00; 1.03]. Furthermore, the higher the social position, the higher the frequency and duration of work uses, respectively *t*(341) = 4.26, *p* < 0.001, IRR = 1.02, 97.5% CI = [1.01; 1.03] and *t*(333) = 2.87, *p* = 0.004, IRR = 1.06, 97.5% CI = [1.02; 1.09]. However, contrary to the hypothesis, the higher the social position, the less participants tent to use it for school work, *t*(341) = −1.88, *p* = 0.060, IRR = 0.99, 97.5% CI = [0.99; 1.00], and the lower the duration of this school work oriented use, *t*(333) = −3.60, *p* < 0.001, IRR = 0.99, 97.5% CI = [0.98; 0.99].

Similar trends emerged concerning the frequency and duration of use during the lockdown for leisure activities: The higher the social position, the higher the frequency and duration of leisure use, respectively, *t*(341) = 2.48, *p* = 0.013, IRR = 1.01, 97.5% CI = [1.00; 1.02] and, *t*(335) = 1.88, *p* = 0.061, IRR = 1.02, 97.5% CI = [0.99; 1.04]. Similarly, the higher the social position, the more participants used their computer for work, *t*(333) = 7.72, *p* < 0.001, IRR = 1.24, 97.5% CI = [1.18; 1.32]. Contrariwise, during the lockdown, duration of use for schoolwork did not depend on social position, *p* = 0.769.

### Perception of Homeschooling Ability

Results indicated that the higher the social position, the higher the homeschooling self-efficacy, *t*(358) = 3.51, *p* < 0.001, IRR = 1.01, 97.5% CI = [1.00; 1.01] and the lower the fear of academic failure, *t*(358) = −4.42, *p* < 0.001, IRR = 0.99, 97.5% CI = [0.98; 0.99].

### Homeschooling Activities

The higher the social position, the lower parents reported helping their children with their homework in normal times, *Z* = −3.12, *p* = 0.002, OR = 0.98, 97.5% CI = [0.97; 0.99]. Furthermore, for those who did help with homework, the higher the social position, the lower the frequency of such help (marginal), *t*(272) = −1.91, *p* = 0.057, IRR = 0.99, 97.5% CI = [0.99; 1.00]. Social position did not impact the probability to engage in homeschooling during the lockdown, *p* = 0.507 nor the frequency of homeschooling, *p* = 0.471. Nevertheless, the higher the social position, the lower the time spent doing homeschooling, *t*(347) = −3.14, *p* = 0.002, IRR = 0.99, 97.5% CI = [0.99; 1.00].

The probability to receive resources from teachers did not depend on social position, *p* = 0.971, nor did the probability to complete the exercises received, *p* = 0.909 or the probability of working on new concepts, *p* = 0.294. However, the higher the social position, the higher the probability of knowing complementary resources (e.g., educative websites or programs), *Z* = 2.91, *p* = 0.004, OR = 1.01, 97.55% CI = [1.00; 1.02], although the frequency of use did not depend on social position, *p* = 0.267.

### Other (Profitable and Unprofitable) Activities During Lockdown

Concerning profitable activities, the higher the social position, the more parents tend to support their children creative activities (marginal), *Z* = 1.94, *p* = 0.052, OR = 1.01, 97.5% CI = [0.99; 1.02], and sport activities and the higher the frequency of such activities, respectively *Z* = 3.55, *p* < 0.001, OR = 1.02, 97.5% CI = [1.01; 1.03] for probability and *t*(298) = 3.41, *p* = 0.001, IRR = 1.01, 97.5% CI = [1.00; 1.01] for frequency.

Finally, although autonomous reading time did not depend on social position during normal time, *p* = 0.347; during lockdown, the higher the social position of the parents, the more children spent time reading in autonomy (marginal), *t*(277) = 1.72, *p* = 0.087, IRR = 1.00, *97.5% CI = [0.*99; 1.01]. Furthermore, the higher their social position, the more parents spent time reading to their children, *t*(309) = 3.63, *p* < 0.001, IRR = 1.01, 97.5% CI = [1.00; 1.01].

Concerning educationally unprofitable activities, the higher the parents’ social position, the less children spent time watching television, both in normal times and during lockdown, respectively *t*(358) = −4.37, *p* < 0.001, IRR = 0.99, 97.5% CI = [0.99; 1.00] for normal time and *t*(358) = −3.91, *p* < 0.001, IRR = 0.99, 95% CI = [0.98; 0.99] for lockdown.

## Discussion

School closures represent a huge challenge for parents, whose role in their children’s learning becomes even more essential than during normal times (Goudeau et al., 2021). In the present research, we argued that, even if all parents were involved in homeschooling during school closures, important variations may emerge depending on the social-class position of the family. Indeed, because of the economic, digital and cultural disparities associated with social class, the lower the parents’ social position, the more they are likely to suffer from both a material and a psychological disadvantage in supporting their children’s learning during lockdown.

First, the results document that, although nearly all respondents had Internet access, the lower the families’ social position, the lower the probability to have a computer, and lower number of owned computers. These results are in line with recent research (Robinson et al., 2020) showing that working-class families experience greater difficulties accessing digital tools (see Legleye and Rolland, 2019; Green, 2020). Hence, accessing the digital tools needed to complete schoolwork during the lockdown may have been particularly challenging in working-class families. Concerning digital uses, contrary to our hypothesis, the higher the social position of the family, the less parents spent time using their computers for schoolwork in normal times. More research is needed to understand this variation, but one possible explanation could be that families with the lower social position, being less comfortable with the academic culture, have a greater need to rely on Internet resources to help their children with their schoolwork than families with higher social position.

Second, in line with our hypothesis, the lower the parents’ social position, the less they felt able to support homeschooling and the more they fear of their children’s academic failure. These findings are consistent with other findings observed in normal times (e.g., Holloway et al., 2016; Tazouti and Jarlégan, 2016). We assume that these differences in perception of schooling ability are due to the fact that working-class families have both fewer digital resources and less familiarity with academic skills and knowledge (Lamont and Lareau, 1988; Gaddis, 2013; Goudeau and Croizet, 2017). In the COVID-19 pandemic context, this unequal familiarity may have enhanced the difficulties encountered by working-class parents to support their children’s work, with further impacts on stress, level of perseverance (Jones and Prinz, 2005), and their real ability to help their children acquire skills and knowledge (Bandura et al., 1996). Interestingly, recent surveys conducted during the lockdown confirm that upper-middle-class parents felt more capable of implementing homeschooling than working-class parents (see Andrew et al., 2020; Bol, 2020; Cullinane and Montacute, 2020).

In line with some past surveys (see Hartas, 2011), our data highlight that in all social classes families were highly involved in the implementation of homeschooling. Interestingly, parents with lower social position reported spending even more time per day homeschooling their children than higher social position ones. Such an observation seems consistent with the fact that they also reported spending more time providing homework help in normal times. As discussed above, this higher level of involvement could be explained by their need for more time to ensure pedagogical continuity as they feel less comfortable with the academic culture and less able to support homeschooling. This interpretation is consistent with the fact that the higher the parents’ position, they more they are likely to know additional educational resources.

Thus, families do not significantly differ regarding their likelihood to engage in homeschooling and monitor their children’s schoolwork. Nevertheless, important disparities emerged concerning the other activities in which they engaged during lockdown, supporting the model of cultural capital disparities among those groups (Bourdieu and Passeron, 1990; Lareau, 2003). Indeed, the higher the parents’ social position, the more they encouraged educationally profitable activities and the less they encouraged educationally unprofitable activities. Indeed, even if these effects would need further investigations, as some of them seem to be driven by child’s age or cohabitation status (which are correlated with social position), higher social position parents more often implemented reading, creative activities, and sports than lower social position ones, who were more likely to have their children watch television.

Based on these results, it seems reasonable to predict that one consequence of school closures could be the widening of the social class achievement gap. This prediction needs further investigation, but is already indirectly supported by research documenting the existence of a summer learning loss (e.g., Cooper et al., 1996; Stewart et al., 2018). This research demonstrated that the social class achievement gap that exists during the school year tends to grow during school breaks, particularly during summer holidays. More importantly, recent research shows that school closure has, indeed, enhanced the social class achievement gap during the first wave of the pandemic (Andreu et al., 2020; Engzell et al., 2020, for a synthesis, see Goudeau et al., 2021). Since academic success is usually claimed to be “meritocratic” (Mijs, 2016; Darnon et al., 2018; Kuppens et al., 2018) and since it subsequently determines future occupations in society, the school system not only contributes to the reproduction of social inequalities but also to their legitimization (Darnon et al., 2018). Thus, the specific situation of lockdown may even accentuate *in fine* this process and the role of education in sustaining future social inequalities (Bourdieu and Passeron, 1990; Goudeau et al., 2017).

Some limitations of the present study must be noted. First, our sample is unbalanced, probably in part due to the recruitment method (i.e., the Internet), with more parents possessing a high social position than lower social position ones. Thus, replications using others methods of recruitment (e.g., questionnaire transferred through teachers), with larger, more class-balanced samples are necessary. Moreover, the present study documented different practices during the lockdown known to impact academic achievement. However, we did not measure academic achievement. In addition, this research was conducted during lockdown, but it is difficult to define the specificity of this period compared to other normal (pre-COVID) periods. For these reasons, we believe a longitudinal study, comparing practices and academic achievement during normal time to those during lockdown, would complement the present findings. Similarly, the present research is cross-sectional and thus, causality cannot be established. Manipulating the salience of lockdown could represent an interesting follow-up of the present study. The effect of social class should be particularly pronounced in contexts in which lockdown is salient, as compared to more neutral contexts.

The COVID pandemic has fundamentally changed the way we live, travel, and interact as well as learn and teach. One consequence of this pandemic has been school closures. From a purely medical perspective, such closures appear both necessary and inevitable, yet we point out the important consequences they may have in terms of children’s learning and achievement and, more largely, academic inequalities. In particular, our results document for the first time the gap that exists in family practices according to social class when schools are closed, thereby highlighting an important impact of lockdowns—namely, the risk of drastically increasing social class educational disparities. By making learning rely more heavily on parents, school closures not only increase the risk of parental stress and burnout (Griffith, 2020; Marchetti et al., 2020; Spinelli et al., 2020), they create very uneven learning situations among children. This represent a very risky situation, particularly if school closures last for several months. In such a situation, national policies providing both economic (e.g., providing the necessary digital equipment) and academic support (e.g., setting up remedial courses) when dealing with such unprecedented situations are necessary to ensure that no child is left behind. Technology-assisted interventions, for example, are particularly efficient to increase the effects of parenting programs during the pandemic amongst socially disadvantaged families (Harris et al., 2020). Similarly, the adaptation of home-based interventions (e.g., EDI model, Bann et al., 2016) could limit the observed disparities by providing parents interactive learning activities that are beneficial to their child’s cognitive development and which could be implemented at home during school closures. It is imperative to anticipate and prevent these phenomena as the whole world is currently experiencing the third wave of the COVID-19 pandemic.

## Data Availability Statement

The datasets presented in this study can be found in online repositories. The names of the repository/repositories and accession number(s) can be found below: https://osf.io/nqdy9/?view_only=a929f02b25d2406d9abad64fa2edefb2.

## Ethics Statement

Ethical review and approval was not required for the study on human participants in accordance with the local legislation and institutional requirements. The patients/participants provided their written informed consent to participate in this study.

## Author Contributions

CS contributed to the conception and design of the study, collected the data and performed the statistical analyses, and wrote the first draft of the manuscript. SG contributed to the conception and design of the study. AS organized the database and performed the statistical analyses. CD contributed to the conception and design of the study. All authors contributed to the manuscript revision, read and approved the submitted version.

## Conflict of Interest

The authors declare that the research was conducted in the absence of any commercial or financial relationships that could be construed as a potential conflict of interest.

## Publisher’s Note

All claims expressed in this article are solely those of the authors and do not necessarily represent those of their affiliated organizations, or those of the publisher, the editors and the reviewers. Any product that may be evaluated in this article, or claim that may be made by its manufacturer, is not guaranteed or endorsed by the publisher.

## Footnotes

1 Social class is mainly assessed using income, educational level and/or occupation. Thus, “Upper-middle-class families” refers to the families in the advantaged range of these indicators (i.e., high-income level, university education level and/or prestigious occupations such as lawyers or researchers). On the contrary, “working-class families” refers to the disadvantaged families on these indicators (i.e., low-income level, no university degree, less prestigious occupations such as blue-collar workers).

2 When they had several children, participants were asked to answer all the questions concerning their practices for the youngest child attending preschool or elementary school.

3 Participants were asked to answer for themselves and for their partner. A composite score was created by averaging the response for the participant and the response for their partner. The reading time score thus corresponds the average time spent reading to the child by one parent.
