# Supplementary material for: A Two-Sided Lockdown? Social Class Variations in the Implementation of Homeschooling During the COVID-19 Lockdown
Source: Front Psychol. 2021 Oct 27;12:670722. doi: 10.3389/fpsyg.2021.670722 (PMC8579058; doi:10.3389/fpsyg.2021.670722)
Supplement: Supplementary file 1 [file Data_Sheet_1.pdf]

Supplementary Material

Table S1.

Measures and Modalities

**Variable**	**Nature**	**Modalities**
	**Digital equipment and uses**	
	Digital equipment	
Own a computer	Categorical	0 = no; 1 = yes
Number of computers	Continuous	*Number*
Internet access	Categorical	0 = no; 1 = yes
High speed Internet access	Categorical	0 = no; 1 = yes
Own a printer	Categorical	0 = no; 1 = yes
	Uses of computer(s) in normal times	
Frequency of leisure activities	Continuous	From 0 (never) to 4 (several times a day)
Duration of leisure activities	Continuous	*Number of hours a week*
Frequency of work	Continuous	From 0 (never) to 4 (several times a day)
Duration of work	Continuous	*Number of hours a week*
Frequency of schoolwork	Continuous	From 0 (never) to 4 (several times a day)
Duration of schoolwork	Continuous	*Number of hours a week*
	Uses of computer(s) since the beginning of lockdown	
Frequency of leisure activities	Continuous	From 0 (never) to 4 (several times a day)
Duration of leisure activities	Continuous	*Number of hours a week*
Frequency of work	Continuous	From 0 (never) to 4 (several times a day)
Duration of work	Continuous	*Number of hours a week*
Frequency of schoolwork	Continuous	From 0 (never) to 4 (several times a day)
Duration of schoolwork	Continuous	*Number of hours a week*
	**Perception of homeschooling ability**	
Self-efficacy (3 items)	Continuous	From 1 (totally disagree) to 7 (totally agree)
Fear of failure (3 items)	Continuous	From 1 (totally disagree) to 7 (totally agree)
	**Homework help during normal times**	
Provide homework help (HH)	Categorical	0 = no; 1 = yes
Frequency of HH	Continuous	From 1 to 7 (days per week)
Duration of HH (per day)	Continuous	*Number of hours a day*
	**Homeschooling activities**	
Do homeschooling (HS)	Categorical	0 = no; 1 = yes
Frequency of HS	Continuous	From 1 to 7 (days per week)
Duration of HS (per day)	Continuous	*Number of hours a day*
Reception of resources	Categorical	0 = no; 1 = yes
Completion of exercises	Continuous	From 1 (none) to 3 (all)

Knowledge of complementary resources (CR)	Categorical	0 = no; 1 = yes
Frequency of CR use	Continuous	From 0 (never) to 4 (every day)
Work on new concepts	Categorical	0 = no; 1 = yes
**Other (profitable) activities during lockdown**		
Do creative activities (CA)	Categorical	0 = no; 1 = yes
Frequency of CA	Continuous	From 1 (less than one time a week) to 4 (every day)
Do sport activities (SA)	Categorical	0 = no; 1 = yes
Frequency of SA	Continuous	From 1 (less than one time a week) to 4 (every day)
Reading by parents (duration)	Continuous	*Number of hours a week*
Autonomous reading during normal times (duration)	Continuous	*Number of hours a week*
Autonomous reading during lockdown (duration)	Continuous	*Number of hours a week*
**Other (unprofitable) activities**		
Watching television in normal times (duration)	Continuous	From 1 (less than 30 minutes a day) to 5 (more than 3 hours a day)
Watching television during lockdown (duration)	Continuous	From 1 (less than 30 minutes a day) to 5 (more than 3 hours a day)
**Socio-demographic (for respondent, then for partner)**		
Gender	Categorical	Woman; Man; Non-binary person
Age	Continuous	*Number*
Number of children	Continuous	*Number*
Child's age	Continuous	*Number*
Child's gender	Categorical	-0.5 = girl; 0.5 = boy
Child's grade	Ordinal	From 1st year of preschool to 5th year of elementary school
Socio-professional category	Categorical	24 INSEE categories (see https://www.insee.fr/fr/information/2497952)
Occupation	Qualitative	*Text*
Education level	Continuous	From 1 (high school) to 6 (PhD or more)
Continuity of work during lockdown	Categorical	-0.5 = no; 0.5 = yes
Type of work	Categorical	-0.5 = part-time; 0.5 = full-time
Hours of work per week	Continuous	*Number of hours a week*
Workplace	Categorical	1 = full remote work; 2 = partial remote work; 3 = full onsite work
Evolution of workload during lockdown	Continuous	From 1 (decrease drastically) to 7 (increase drastically)
Number of books in the house	Continuous	*Number*
Reading time per week	Continuous	*Number of hours a week*

Extra-curricular activities (ECA)	Categorical	0 = no; 1 = yes
Number of ECA	Continuous	*Number (based on ECA list)*

Table S2.

Social position index values, translated from Rocher (2016)

SCP	Label	Mother	Father
**10**	Farmers-operators [Agriculteurs exploitants]	99	104
**21**	Craftsman [Artisans]	95	95
**22**	Merchants and similar [Commerçants et assimilés]	101	102
**23**	Managers of compagnies with ten or more employees [Chefs d'entreprise de dix salaries ou plus]	128	140
**31**	Liberal occupations and assimilated [Professions libérales et assimilés]	155	158
**33**	Public service executives [Cadres de la fonction publique]	154	148
**34**	Faculty teacher, scientific position [Professeurs, professions scientifiques]	163	160
**35**	Information, arts and entertainment positions [Professions de l'information, des arts et des spectacles]	143	147
**37**	Administrative and commercial managers [Cadres administratifs et commerciaux d'entreprise]	145	148
**38**	Engineers and technical managers of companies [Ingénieurs et cadres techniques d'entreprises]	164	157
**42**	Primary School teachers and similar [Professeur des écoles, instituteurs et assimilés]	154	149
**43**	Intermediate occupations in health and social work [Professions intermédiaires de la santé et du travail social]	120	125
**44**	Clergy, religious [Clergé, religieux]	147	132
**45**	Intermediate public service occupations [Professions intermédiaires de la fonction publique]	115	116
**46**	Intermediate occupations in companies [Professions intermédiaires en entreprise]	125	123
**47**	Technicians [Techniciens]	125	115
**48**	Foremen, supervisors [Contremaîtres, agents de maîtrise]	122	109
**52**	Civilian employees and public service officers [Employés civils et agents de service de la fonction publique]	95	95
**53**	Police and military [Policiers et militaires]	110	115
**54**	Business administrative employees [Employés administratifs d'entreprises]	113	115
**55**	Commercial employees [Employés de commerce]	88	93
**56**	Direct personal service workers [Personnels des services directs aux particuliers]	82	86
**61**	Qualified workers [Ouvriers qualifiés]	78	79
**66**	Unqualified workers [Ouvriers non qualifiés]	65	64
**69**	Agricultural workers [Ouvriers agricoles]	67	60

**71**	Former farmers-operators [Anciens agriculteurs exploitants]	76	84
**72**	Former craftsmen, merchants, and managers of companies [Anciens artisans, commerçants, chefs d'entreprise]	101	93
**73**	Former managers and intermediate occupations [Anciens cadres et professions intermédiaires]	129	123
**76**	Former employees and workers [Anciens employés et ouvriers]	97	59
**81**	Unemployed never worked [Chômeurs n'ayant jamais travaillé]	51	54
**82**	Various persons without professional activities [Personnes diverses sans activités professionnelle]	75	61
**99**	Not filled in: unknown or not applicable [Non renseignée : inconnue ou sans objet]	91	70

Note. This index is standardized, at a mean of 100 and a standard deviation of 30.

Table S3

Results of model 2, integrating covariates (respondent's age, gender, number of children and cohabitation status) for Digital Equipment

	Social position index	Respondent's age	Respondent's gender	Number of children	Cohabitation status
**Own a computer**	1.02 [1.00;1.05] *	1.01 [0.90;1.14]	1.32 [0.19;9.05]	0.72 [0.37;1.39]	4.24 [1.14;15.83] *
**Number of computers**	1.01 [1.00;1.01] ***	1.04 [1.01;1.07] **	1.39 [0.97;1.98] t	0.99 [0.86;1.14]	1.89 [1.42;2.49] ***
**High-speed Internet**	1.02 [1.01;1.03] **	0.93 [0.87;0.99] *	2.19 [0.85;5.62]	0.84 [0.59;1.19]	0.71 [0.27;1.82]
**Own a printer**	1.00 [0.995;1.01]	1.02 [0.97;1.07]	1.13 [0.62;2.07]	1.15 [0.84;1.58]	1.85 [0.96;3.44] t

Note. Odd Ratios [97.5% Confidence Interval] are reported for categorical variables while IRR [97.5% Confidence Interval] are reported for continuous variable. Statistical differences are highlighted as follows: t p < .10; * p < .05; ** p < .01; ***p < .001

Table S4

Results of model 2, integrating covariates (respondent's age, gender, number of children, continuity of work, cohabitation status, child's age and gender) for Self-efficacy, Fear of failure, Homeschooling and Other Activities

	Social position index	Respondent's age	Respondent's gender	Number of children	Continuity of work	Cohabitation status	Child's age	Child's gender
			**Perception of homeschooling ability**					
**Self-efficacy**	1.01 [1.00; 1.02] **	1.02 [0.99; 1.04]	0.88 [0.66; 1.19]	0.93 [0.76; 1.13]	0.92 [0.66; 1.28]	0.91 [0.60; 1.39]	0.96 [0.90; 1.03]	1.01 [0.75; 1.38]
**Fear of academic failure**	0.99 [0.98; 0.997] **	0.95 [0.91; 0.98] **	0.81 [0.59; 1.11]	1.10 [0.92; 1.32]	1.04 [0.73; 1.49]	0.93 [0.56; 1.54]	1.13 [1.04; 1.22] **	0.97 [0.77; 1.23]
			**Homework help during normal times**					
**Provide homework help (HH)**	0.98 [0.96; 1.00] *	1.01 [0.94; 1.09]	2.00 [0.86; 4.66]	1.75 [1.14; 2.68] *	0.69 [0.32; 1.47]	0.16 [0.04; 0.73] *	1.98 [1.58; 2.49] ***	0.49 [0.28; 0.86] *
**Frequency of HH**	1.00 [0.99; 1.00]	1.02 [0.99; 1.06]	0.70 [0.44; 1.11]	1.28 [0.97; 1.69]	0.96 [0.65; 1.42]	0.82 [0.48; 1.40]	1.04 [0.93; 1.17]	1.25 [0.77; 2.01]
			**Homeschooling during lockdown**					
**Do homeschooling (HS)**	1.02 [0.99; 1.05]	1.03 [0.85; 1.25]	2.64 [0.11; 6.62]	0.51 [0.16; 1.66]	0.03 [0.00; 4.47]	3.50 [0.43; 2.85]	0.69 [0.43; 1.12]	1.76 [0.41; 7.54]
**Frequency of HS**	1.00 [0.99; 1.00]	1.00 [0.98; 1.01]	1.11 [0.90; 1.37]	1.04 [0.91; 1.19]	1.13 [0.91; 1.41]	1.16 [0.86; 1.58]	1.05 [1.01; 1.10] *	1.06 [0.83; 1.37]

**Duration of HS (per day)**	0.99 [0.99; 1.00] *	1.01 [0.98; 1.03]	0.86 [0.67; 1.10]	1.30 [1.14; 1.48] ***	1.01 [0.77; 1.32]	0.83 [0.61; 1.15]	1.13 [1.06; 1.21] ***	0.92 [0.77; 1.10]
**Completion of exercises (none vs. some/every)**	0.98 [0.96; 1.01]	0.89 [0.79; 1.00] *	0.63 [0.20; 2.67]	1.29 [0.62; 2.68]	2.73 [0.85; 8.77] t	2.55 [0.75; 8.73]	1.08 [0.82; 1.43]	1.08 [0.38; 3.07]
**Knowledge of complementary resources (CR)**	1.01 [1.00; 1.02] *	1.05 [1.00; 1.11] t	0.39 [0.22; 0.68] ***	1.03 [0.75; 1.41]	1.23 [0.69; 2.20]	0.38 [0.16; 0.89] *	0.95 [0.84; 1.08]	1.11 [0.72; 1.69]
**Frequency of CR use**	0.99 [0.98; 1.00] *	1.02 [0.97; 1.08]	1.18 [0.70; 1.99]	1.00 [0.79; 1.28]	1.36 [0.78; 2.36]	1.28 [0.72; 2.30]	0.85 [0.75; 0.97] *	1.06 [0.71; 1.56]
**Work on new concepts**	1.01 [1.00; 1.02]	0.98 [0.93; 1.03]	1.21 [0.72; 2.05]	0.92 [0.69; 1.22]	0.72 [0.43; 1.23]	0.83 [0.43; 1.61]	0.84 [0.75; 0.94] **	0.90 [0.60; 1.34]
**Other (profitable) activities during lockdown**								
**Do creative activities (CA)**	1.00 [0.99; 1.01]	1.00 [0.93; 1.07]	1.18 [0.52; 2.69]	1.07 [0.69; 1.67]	0.96 [0.43; 2.16]	1.88 [0.83; 4.26]	0.61 [0.50; 0.73] ***	0.99 [0.55; 1.79]
**Do sport activities (SA)**	1.01 [1.00; 1.02] t	1.03 [0.96; 1.10]	1.00 [0.47; 2.12]	1.18 [0.77; 1.82]	1.39 [0.70; 2.79]	2.86 [1.40; 5.88] ***	1.00 [0.85; 1.17]	1.21 [0.70; 2.19]
**Frequency of SA**	1.00 [1.00; 1.01] *	1.01 [0.99; 1.03]	0.96 [0.80; 1.14]	0.97 [0.88; 1.07]	1.13 [0.93; 1.37]	1.08 [0.86; 1.35]	0.98 [0.94; 1.02]	0.98 [0.83; 1.16]
**Reading by parents (duration)**	1.00 [0.99; 1.01]	1.01 [0.98; 1.05]	1.16 [0.86; 1.58]	0.93 [0.77; 1.11]	0.91 [0.65; 1.26]	1.32 [0.86; 2.04]	0.79 [0.73; 0.86] ***	1.05 [0.82; 1.34]

**Reading in autonomy during normal times (duration)**	1.00 [0.99; 1.01]	1.01 [0.99; 1.03]	1.37 [1.04; 1.79] *	0.99 [0.87; 1.12]	0.96 [0.76; 1.21]	0.92 [0.67; 1.27]	1.02 [0.97; 1.07]	1.04 [0.89; 1.22]
**Reading in autonomy during lockdown (duration)**	1.00 [1.00; 1.01]	1.02 [0.99; 1.01]	1.21 [0.90; 1.62]	0.93 [0.77; 1.11]	1.27 [0.93; 1.72]	1.06 [0.73; 1.54]	1.03 [0.96; 1.10]	1.21 [0.91; 1.61]
**Other (unprofitable) activities**								
**Watching television in normal times (duration)**	0.99 [0.99; 1.00] **	0.99 [0.97; 1.01]	1.11 [0.89; 1.39]	1.03 [0.92; 1.16]	0.89 [0.69; 1.14]	0.88 [0.66; 1.16]	1.06 [1.01; 1.11] *	0.96 [0.84; 1.10]
**Watching television during lockdown (duration)**	0.99 [0.99; 1.00] **	0.99 [0.96; 1.02]	1.11 [0.81; 1.52]	1.02 [0.85; 1.22]	1.06 [0.78; 1.45]	0.65 [0.45; 0.93] *	1.07 [1.00; 1.15] t	0.90 [0.70;1.15]

Note. Odd Ratios [97.5% Confidence Interval] are reported for categorical variables while IRR [97.5% Confidence Interval] are reported for continuous variable. Statistical differences are highlighted as follows: t p < .10; * p < .05; ** p < .01; ***p < .001
